# Knowledge about glaucoma among adults in Africa: a systematic review

**DOI:** 10.1186/s12886-024-03333-9

**Published:** 2024-02-14

**Authors:** Wondwossen Yimam, Tamrat Anbesaw, Muhammed Seid, Prem Kumar, Hawa Wolie

**Affiliations:** 1 Department of Comprehensive Nursing, College of Medicine and Health Sciences (CMHS), Wollo University (WU), Dessie, Ethiopia; 2 Department of Psychiatry, CMHS, Dessie, WU Ethiopia; 3 Department of Adult Health Nursing, CMHS, Dessie, WU Ethiopia

**Keywords:** Africa, Glaucoma, Knowledge, Systematic review

## Abstract

**Background:**

Africa is one of the continents with a substantial burden of glaucoma, where it is approximately twice as common as it is worldwide. If left untreated, glaucoma can cause blindness and permanent vision loss if it is not addressed promptly. Good knowledge is essential for preventing glaucoma’s irreversible blindness and ocular damage. However, no systematic review has been done to report the pooled percentage of adults in Africa who have good knowledge about glaucoma. This study aimed to estimate the level of knowledge about glaucoma in Africa and its determinants.

**Methods:**

The study followed the PRISMA guidelines for systematic review. Seven electronic databases which include PubMed/MEDLINE, Web of Science, PROQUEST (PQDT), CINAHL (EBSCO), Google Scholar, African Journal Online, and WHO HINARI databases were searched from January 1, 2013, to July 31, 2023for studies conducted with a focus on the knowledge about glaucoma among adults in Africa. The quality of the final articles was assessed using the Joanna Briggs Institute quality assessment tool for cross-sectional studies.

**Results:**

In the present systematic review, 2781 articles were initially identified and evaluated. Of these, eight studies that met the inclusion criteria were included in the final analysis. In this review, the proportion of knowledge about glaucoma among adults in Africa was low. Educational status, family history of glaucoma, occupation, being a male, and having a history of eye examination were the main determinants of good knowledge about glaucoma among adults in Africa.

**Conclusion:**

The systematic review found that only a few had good knowledge about glaucoma. Education campaigns and eye exam promotions are recommended to enhance awareness.

**Trail registration:**

This systematic review was registered on 30/07/2023 with PROSPERO ID: CRD42023430723.

**Supplementary Information:**

The online version contains supplementary material available at 10.1186/s12886-024-03333-9.

## Background

Glaucoma is a chronic, progressive, degenerative optic nerve disorder causing vision loss and blindness. Early detection and treatment are crucial for controlling and preventing glaucoma-induced blindness. Good knowledge of glaucoma screening is a vital step to control and prevent glaucoma-induced blindness [1]. Glaucoma is the second leading cause of blindness after cataracts and the leading cause of irreversible blindness in the world [2, 3]. According to the global association report, more than 78 million populations live with glaucoma and about 90% of glaucoma is undetected in developing countries. The global prevalence of glaucoma was estimated at 2.4% in 2021 [4]. In Africa (2021), glaucoma prevalence was estimated at 4.2% for primary open-angle glaucoma (POAG) and 1.09% for primary angle-closure glaucoma (PACG) [3, 4]. Various studies from different regions of the world have revealed the varying levels of glaucoma knowledge among the population.

The World Health Organization recommends glaucoma screenings every 2–4 years for individuals under 40, 2–3 years for those 40–60, and 1–2 years for those over 60. However, the level of knowledge for glaucoma screening varies throughout African nations [2]. Although a few cross-sectional studies have been conducted previously in different parts of Africa on knowledge about glaucoma, the pooled percentage of good knowledge about glaucoma among adults in Africa is unknown. Additionally, Inconsistent proportions of both good knowledge and determining factors have also been observed by several researchers. This may be a result of regional variations in sociocultural, educational, and health service access among the study individuals. The goal of this systematic review was to examine the pooled percentage of good knowledge about glaucoma and the determinant factors influencing it.

## Data source and search strategy

This study used both published and unpublished literature that evaluated the percentage of good knowledge of glaucoma among African adults as data sources. The review protocol has been registered in the International Prospective Register of Systematic Review (PROSPERO) (CRD42023430723) [16] and follows the Cochrane Manual’s recommendations (version 6.3) [15] for conducting systematic intervention reviews. The report adhered to the guidelines of The Preferred Reporting Items for Systematic Reviews and Meta-Analyses Protocols (PRISMA flow diagram [17] to ensure a high-quality report. The search strategy was devised using the Population Intervention Comparison and Outcome (PICO) search guide. For published articles, a comprehensive search of online databases such as PubMed/MEDLINE, Web of Science, PROQUEST (PQDT), CINAHL (EBSCO), Google Scholar, African Journal Online, and WHO HINARI databases were used. Unpublished literature such as agency reports, governmental articles, and academic thesis were extensively searched from the online library of governmental and academic institutions in Ethiopia from Addis Ababa University and the AfroLib databases to access the unpublished literature of African studies. The search was carried out using the following search keywords and medical subject headings: ((“knowledge“[MeSH Terms] OR “knowledge“[All Fields] OR “knowledge s“[All Fields] OR “knowledgeability“[All Fields] OR “knowledgeable“[All Fields] OR “knowledgeably“[All Fields] OR “knowledges“[All Fields] OR “awareness*“[All Fields] OR “knowledge“[MeSH Terms] OR “health knowledge, attitudes, practice“[MeSH Terms]) AND (“2013/07/18 00:00”:“3000/01/01 05:00“[Date - Publication] AND “humans“[MeSH Terms]) AND ((“Glaucoma“[MeSH Terms] OR “Glaucoma“[All Fields] OR “glaucomas“[All Fields] OR “glaucoma s“[All Fields] OR “glaucoma screening“[All Fields] OR “Glaucoma“[MeSH Terms] OR “Ocular Hypertension“[MeSH Terms] OR “glaucoma, angle closure“[MeSH Terms] OR “glaucoma, open angle“[MeSH Terms]) AND (“2013/07/18 00:00”:“3000/01/01 05:00“[Date - Publication] AND “humans“[MeSH Terms]))) AND ((y_10[Filter]) AND (humans[Filter])) (Additional file 1).

### Eligibility criteria

#### Inclusion criteria

The main outcome of this study was good knowledge about glaucoma among adults in Africa and measured based on proportion. An odds ratio with a 95% confidence interval was used as an effect measure in this study. The inclusion criteria were the following:-.

- **Study setting**: Studies done in Africa.
- **Study participants**: Adult (human) population (≥ 18 years) from community or institution-based settings.
- **Publication status**: All published and unpublished quantitative studies reported a proportion of knowledge and or OR/RR with 95% CI.
- **Language**: Only studies published in English were included.
- **Types of studies**: Studies that employed observational study design.
- **Publication date**: The authors included articles published between January 1, 2013, and July 31, 2023.

### Exclusion criteria

Reviews, case series, and short communications; editorial, letter to the editor, commentary, perspective, and preprint literature; articles that didn’t report specific outcomes related to knowledge toward glaucoma, articles with unclear measurement for an outcome variable, studies that only reported qualitative findings, studies reported KAP on glaucoma among healthcare workers and glaucoma patients, book chapters, and studies that did not present primary data were excluded.

### Operational definitions

Good or adequate knowledge: Knowledge of glaucoma with the sum of the scores for each participant greater than the mean or ≥ 50% percentage score of the knowledge-related questionnaire.

### Data extraction

Initially, the articles obtained from the selected databases were exported to the EndNote version 8 software packages, and exact duplicates were removed. Then, the EndNote library was shared between two reviewers who independently screened articles by title and abstract for possible inclusion. Then, they reviewed the full-text articles of the selected studies to determine the final inclusion. Using a standardized Excel sheet for data extraction, two authors (WY and MS) independently extracted the data from the full texts of the retained articles. Any disagreements were settled by talking to a third author (TA). Authors, publication year, study design, study setting, country or study area, tool used, participant age, sample size, sampling procedure, outcome, and study quality were all included in the data extraction format (Table 1).

**Table 1:** Characteristic *of selected studies*

Authors	Publication Year	Study Design	Study Setting	Country/ Study Area	Tool	Participants Age (Years)	Sample Size	Sampling Technique	Good Knowledge(n)	Study Quality
Molla I, et al.	2022	CS	Community Based	Ethiopia	Unstandardized	≥ 35 years	230	Multistage	117	9
Ocansey T, et al.	2021	CS	Community Based	Ghana	Unstandardized	≥ 18 years	326	Cluster	99	8
Assavedo C, et al.	2021	CS	Community Based	Benin	Unstandardized	≥ 18 years	548	Not clear	48	8
Osayem J, et al.	2021	CS	Community Based	Nigeria	Unstandardized	≥ 18 years	163	Multistage	54	8
Yenegeta Z, et al.	2020	CS	Community Based	Ethiopia	Unstandardized	≥ 18 years	594	Systematic sampling	100	9
Alemu D, et al.	2017	CS	Community Based	Ethiopia	Unstandardized	≥ 35 years	246	Multistage	122	8
Ibanga, A, et al.	2017	CS	Institution Based	Nigeria	Unstandardized	≥ 18 years	275	Systematic sampling	24	9
Ogbonnaya C, et al.	2016	CS	Community Based	Nigeria	Unstandardized	≥ 18 years	402	Purposive	27	8

### Quality assessment

Two independent reviewers (TA and PK) conducted a full-text quality assessment for each study in the review. The Joanna Briggs Institute quality assessment tool for cross-sectional studies [5] was used to assess the quality of the studies included in this review. The disagreement between the two reviewers was resolved by discussion with the third team member (HW). Using frequency scales with the responses “yes,” “no,” “unclear,” and “not applicable,” each publication was scored. Based on the total number of positive scores, the overall quality score for each study was assessed. The studies included in the final analysis got a mean quality score of 8.25, ranging from eight to nine, according to the Joanna Briggs Institute quality evaluation checklist. Six other articles were of moderate quality (scores between 6 and 8.75), and two studies were of high quality (scores of 8.75). None of the articles had poor quality (Additional File 2).

### Synthesis of findings

Previous studies used mean and percentage to dichotomize the level of knowledge about glaucoma because of the heterogeneous nature of the review findings; the authors did not compute meta-analysis.

## Results

### Search result

**Fig. 1:**
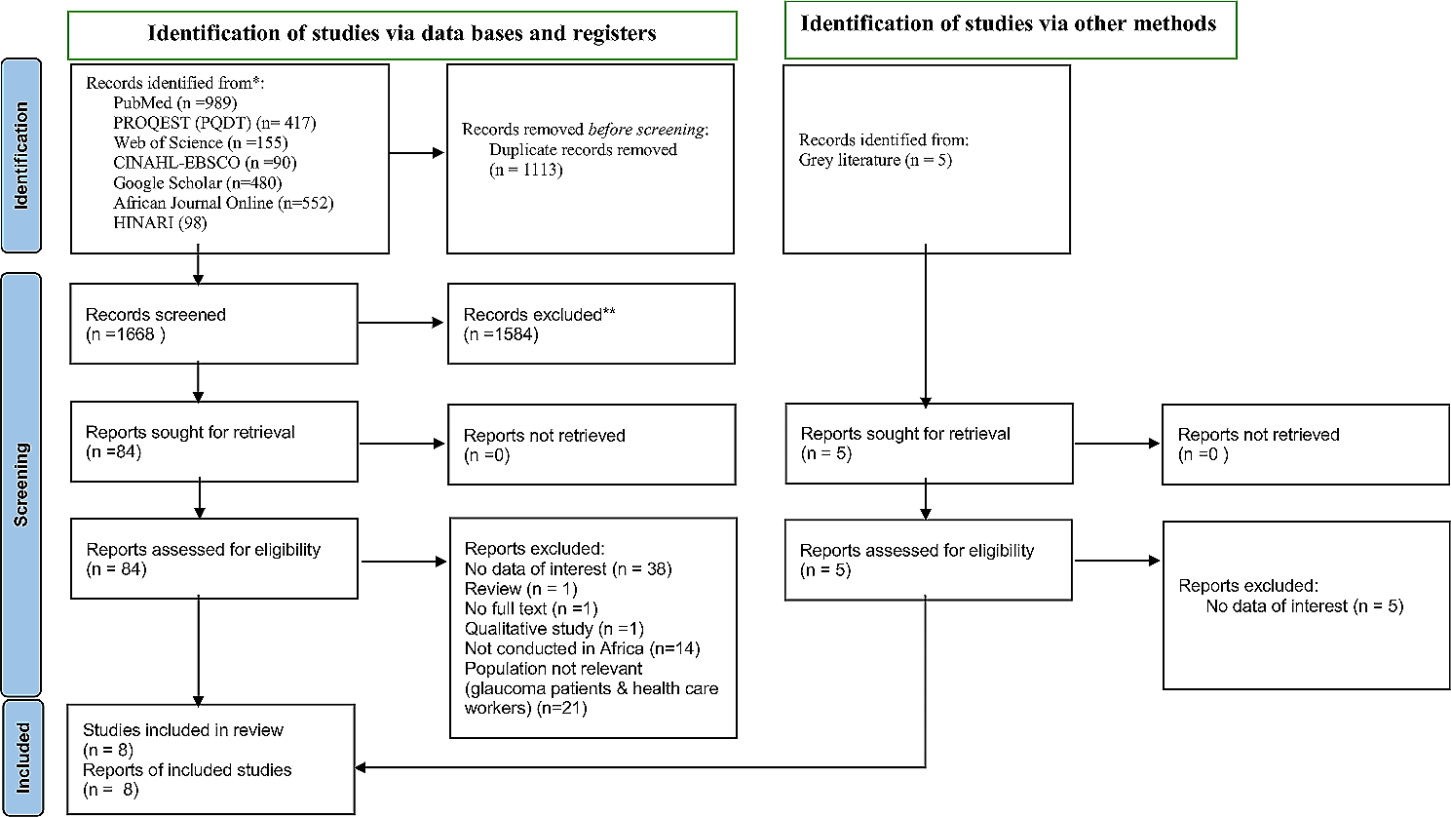
PRISMA flow diagram of included studies

Initially, the articles obtained from the selected databases were exported to the EndNote 8 software package. Then, the EndNote data was shared between two reviewers (WY and MS), who independently screened articles by title and abstract. A total of 2781 records were retrieved from different electronic databases, of which 1113 were duplicates. The titles and abstracts of 1668 articles were assessed, and 1584 articles were removed using EndNote 8 software packages since their titles and abstracts were not in line with the inclusion criteria. Then, after reaching a consensus, a full text of 84 articles was retained for further eligibility analysis by the two independent reviewers, eight of whom were qualified for the present systematic review (Fig. 1).

In this review, a total of 8 articles were included in the final systematic review conducted in four different countries in Africa representing 2784 study subjects. Three studies were from Ethiopia and Nigeria, and two studies were from Benin and Ghana. Of the 8 studies, three were published from 2013 to 2018 years [6–8] and five were published from 2019 to 2023 years [9–13]. Five studies used a sample size of less than 400 [6, 7, 9–11], and three of the studies used a sample size of more than 400 [8, 12, 13]. A small sample size (163) was observed in a study conducted in Ikpoba-Okha, South-South Nigeria, and a large sample size (594) in Gish Abay town, Northwest Ethiopia (Table 1).

### Knowledge about glaucoma and its determinants

A community-based study conducted in Jimma town, southwest Ethiopia determined the level of good knowledge of glaucoma among adults and its determinants, by taking a sample of 230 participants from Jimma town aged 35 years and above. Findings demonstrated that the level of good knowledge of glaucoma was found to be 50.9%. This study concluded that only half of the participants had a good knowledge level of glaucoma among Jimma residents. Educational status [(AOR = 6.07, 95% CI:(2.06, 17.87)] and ever had eye examination [(AOR = 3.58, 95% CI:(2.01, 6.40)] were the associated factors with knowledge about glaucoma [9]. Likewise, a cross-sectional study was conducted in Gondar Town, Northwest Ethiopia with 246 adults to determine good knowledge about glaucoma and risk factors. Results showed that the participants possessed a good level of knowledge about glaucoma below average (49.6%, 95%CI: 43.3%, 55%). Level of education (primary [AOR:2.83;1.04,7.71], secondary[AOR:3.45;1.33,9.41], college and above [AOR: 4.86;1.82,12,99] and having a history of eye examination [AOR: 2.61;1.53,4.45] were found to have significantly associated with knowledge about glaucoma [6].

Similarly, in Ikpoba-Okha, south-south Nigeria a community-based study investigated 163 adults aged ≥ 18 years to determine their knowledge about glaucoma and its predictors. The findings revealed that only 38% of the participants had a good level of knowledge about glaucoma. Respondents in the age group of 30–39 years (*p* < 0.001) and those with a tertiary level of education (*p* < 0.001) were significantly associated with knowledge about glaucoma. The conclusion of the study reported that the majority of respondents had no good knowledge of glaucoma [10].

In rural and urban residents of Ghana, a population-based cross-sectional survey was conducted involving 326 adults aged ≥ 18 years of age. The finding indicated that only a few study participants (30.4%) had good knowledge about glaucoma (30.4%, 95% CI = 25.4–35.4). The conclusion of the study reported that the main factors of glaucoma knowledge were educational status and previous eye examination [11]. Similarly, a community-based study among adults (≥ 18 years) in Gish Abay town, Northwest Ethiopia revealed that good knowledge was demonstrated in 16.8% [95% CI; 14.0, 19.9] of participants. Educational status: primary education [AOR; 2.89: 1.41, 5.90], secondary education [AOR; 3.03: 1.47, 6.24] college and above [AOR; 5.18: 2.21, 12.13], history of eye examination [AOR; 6.52: 3.37, 12.63]; family history of glaucoma [AOR; 12.08: 4.13, 35.30] and higher income level [AOR; 3.11: 1.55, 6.25] were positively associated with good knowledge of glaucoma. The conclusion of the study stated that the proportion of good knowledge of glaucoma was low [13].

An Institution-based study was conducted in a rural area of Cross River State, Nigeria among 275 adults to determine the level of knowledge about glaucoma and its risk factors. Results stated that only 8.7% of participants knew about glaucoma. Average monthly income (X2 = 15.771, *P* = 0.003) and previous eye checks (X2 = 7.565, *P* = 0.023) were predictors of good knowledge about glaucoma. The conclusion of the study described that the level of knowledge of glaucoma was poor in the population and average monthly income and previous history of eye checks were significantly associated with knowledge [7].

A community-based study was conducted among 402 adult residents in a Rural Community of Ebonyi State, Nigeria, aged ≥ 18 years. Results indicated that only 27(6.3%) of the respondents had good knowledge about glaucoma. In this study, higher education (> secondary) (X2 = 7.30; *p* = 0.007) and being male (29.3% men vs. 14.9% women; X2 = 12.27; *p* < 0.001) were predictors of knowledge about glaucoma. The conclusion of the study described that the proportion of good knowledge of glaucoma was low [8]. In the same way, a study was conducted in Northern Benin among 548 adults to determine the level of knowledge about glaucoma and its risk factors. Results specified that only 1.24% of participants had a good knowledge of glaucoma. Education (high school and university levels) and occupation were significantly associated with knowledge about glaucoma. The conclusion of the study described that the proportion of good knowledge of glaucoma was alarmingly low [12] (Table 2).

**Table 2:** Summarized study findings

Authors/Year/Title	Findings	Conclusion
Molla I, et al.,2022 (Glaucoma and associated factors among adults in Jimma town, southwest Ethiopia)	1. The proportion of good knowledge towards glaucoma among adult was 50.9% (95% CI 44.3%, 57.8%). 2. Educational status [(AOR = 6.07, 95% CI: (2.06, 17.87)], and ever had eye examination [(AOR = 3.58, 95% CI: (2.01, 6.40)], were the associated factors with knowledge of glaucoma.	1. Half of them had good knowledge of glaucoma. 2. Educational status and eye examination were associated factors with good knowledge of glaucoma.
Ocansey T,et al.,2021 (Socio-demographic factors modify awareness, knowledge, and perceived risk of glaucoma in rural and urban residents in Ghana: a population based survey)	1. Good knowledge was demonstrated in 99 (30.4%, 95% CI = 25.4–35.4) participants about glaucoma. 2. Educational status [(AOR = 1.35, 95% CI: (1.2, 1.49)], and ever had eye examination [(AOR = 1.96, 95% CI: (1.35, 2.52)], were the associated factors with knowledge of glaucoma.	1. Only few had good knowledge of glaucoma. 2. The main factors of glaucoma knowledge were education and previous eye examination.
Assavedo C,et al.,2021 (Knowledges, Attitudes and Practices Related to Primitive Open Angle Glaucoma in The Adult Population in Northern Benin)	1. Only 1.24% had a good knowledge of glaucoma.	1. The level of knowledge of glaucoma in the adult population in Benin is alarmingly low 2. Education (high school and university levels) and occupation were significantly associated with glaucoma.
Osayem J,et al.,2021 (Awareness and knowledge on glaucoma in Ikpoba-Okha, south-south Nigeria)	1. 38% of the respondents had good knowledge of glaucoma 2. Respondents in the age group of 30–39 years (*p* < 0.001) and those with tertiary level of education (*p* < 0.001) had higher awareness of glaucoma.	1. The majority of respondents had no good knowledge of glaucoma 2. Education and age group of 30–39 years were significantly associated with glaucoma.
Yenegeta Z,et al.,2020 (Knowledge of glaucoma and associated factors among adults in Gish Abay town, Northwest Ethiopia)	1. The proportion of good knowledge was demonstrated in 16.8% [95% CI; 14.0, 19.9] 2. Educational status: primary education [AOR; 2.89: 1.41, 5.90], secondary education [AOR; 3.03: 1.47, 6.24] college and above [AOR; 5.18: 2.21, 12.13], history of eye examination [AOR; 6.52: 3.37, 12.63]; family history of glaucoma [AOR; 12.08: 4.13, 35.30] and higher income level [AOR; 3.11: 1.55, 6.25] were positively associated with good knowledge of glaucoma.	1. The proportion of good knowledge of glaucoma was low 2. Higher educational status, positive family history of glaucoma; eye examination and higher income level were significantly associated with knowledge of glaucoma.
Alemu D,et al.,2017 (Awareness and knowledge of glaucoma and associated factors among adults: a cross sectional study in Gondar Town, Northwest Ethiopia)	1. Good knowledge was demonstrated in 49.6% (95% CI: 43.3%, 55%) of participants. 2. Level of Education (primary [AOR:2.83;1.04,7.71], secondary [AOR:3. 45;1.33,9.41], college and above [AOR: 4.86;1.82,12,99] and having eye examination [AOR: 2.61;1.53,4.45] were significantly associated with knowledge.	1. The proportion of good knowledge of glaucoma was low inadequate 2. Education and having eye examination were significantly associated with knowledge.
Ibanga, A,et al.,2017 (Glaucoma awareness and knowledge among people attending ophthalmic outreach services in a rural area of Cross River State, Nigeria)	1. Only 8.7% had good knowledge of glaucoma 2. Average monthly income (**χ**^**2**^ = 15.771, *P* = 0.003) and previous eye checks (**χ**^**2**^ = 7.565, *P* = 0.023).	1. The level of knowledge of the glaucoma was poor in the population. 2. Average monthly income and previous eye checks were significantly associated with knowledge.
Ogbonnaya C,et al.,2016 (Glaucoma Awareness and Knowledge, and Attitude to Screening, in a Rural Community in Ebonyi State, Nigeria)	1. Only 27(6.3%) of the respondents had good knowledge about glaucoma 2. Education (> secondary) (**χ**^**2**^ = 7.30; *p* = 0.007) and being male (29.3% men vs. 14.9% women; **χ**^**2**^ = 12.27; *p* < 0.001) were predictors of knowledge.	1. The proportion of good knowledge of glaucoma was low. 2. Higher educational status and gender (male) were significantly associated with knowledge of glaucoma.

AOR: Adjusted Odds Ratio; CI: Confidence Interval; P: *p*-value; **χ**^**2**^: Chi-square

## Discussion

Africa is one of the most affected continents on the globe by glaucoma. The prevalence of glaucoma is almost twofold higher than the global prevalence; hence, assessment of the knowledge level of glaucoma among adults in Africa is vital to increase the health-seeking behavior of adults for an eye examination. To our knowledge, this is the first study to systematically review and assess the pooled percentage of good knowledge about glaucoma among adults in Africa, which was looked at across 8 studies and involved 2784 adult participants. Studies showed good knowledge about glaucoma among adults in Africa, varying from about 1.24–50.9%.

In all reviewed eight studies, the proportion of participants who knew about glaucoma was below 50.9% [6–13]. A high proportion of knowledge was reported from a study conducted in Ethiopia (50.9%) [9] and a low level of knowledge about glaucoma was detected in Northern Benin (1.24%) [12]. This review revealed that the level of good knowledge about glaucoma among adults in Africa is generally low. The highest proportion of knowledge about glaucoma was found in South India (82%), followed by China (59%), India (41%), and Syria (8%) [14–17].

This difference could be due to sociocultural, access to healthcare, availability of ophthalmic centers, and educational system differences among nations. This finding may have repercussions for healthcare professionals who may wish to think about supporting glaucoma awareness campaigns and educational initiatives to raise awareness and knowledge among adults.

Educational status (higher level) [6–13], having a history of eye examination [6, 9, 11, 13], high-income level [13], a family history of glaucoma [13], age group of 30–39 years [10], gender (being a male) [8] were significant and positive predictors of knowledge about glaucoma among adults in Africa (Table 2). High level of education, a family history of glaucoma, youths (30–39 years), males, and high income were associated with knowledge about glaucoma. This could be because literacy is significantly associated with increased access to knowledge about glaucoma. Having a history of eye examination [6, 9, 11, 13] was also associated with knowledge about glaucoma among adults in four studies. This might be due to access to information by healthcare workers during their visits to health institutions. This finding could have implications for healthcare workers, who may want to consider promoting regular eye exams as a way to increase knowledge about glaucoma among the adult population.

In this systematic review, previous literature failed to use standardized tools. Secondly, all of the included studies were performed only in four countries in Africa, which significantly affects the African representativeness of the estimates. Thirdly, studies published in languages other than English were excluded, and studies could also be missed since we could not use all the databases.

## Conclusion

The proportion of knowledge about glaucoma among adults in Africa was low. Educational status, family history of glaucoma, occupation, being a male, and having a history of eye examination were the main determinants of good knowledge about glaucoma among adults in Africa. Education campaigns and the promotion of eye exams as a strategy to enhance the adult population’s level of good knowledge about glaucoma are recommended.

### Electronic supplementary material

Below is the link to the electronic supplementary material.

Supplementary Material 1

Supplementary Material 2

## Data Availability

The datasets used and/or analyzed during the current study are available from the corresponding author upon reasonable request.
