# Supplementary material for: Knowledge about glaucoma among adults in Africa: a systematic review
Source: BMC Ophthalmol. 2024 Feb 14;24:69. doi: 10.1186/s12886-024-03333-9 (PMC10868007; doi:10.1186/s12886-024-03333-9)
Supplement: Supplementary file 2 — Supplementary Material 2 [file 12886_2024_3333_MOESM2_ESM.pdf]

Authors	Response									Total
	**Q1**	**Q2**	**Q3**	**Q4**	**Q5**	**Q6**	**Q7**	**Q8**	**Q9**	
Molla I , et al	Y	Y	N	Y	Y	Y	Y	Y	Y	8
Ocansey T,et al	Y	Y	Y	Y	Y	Y	Y	Y	N	8
Assavedo C,et al	Y	N	Y	Y	Y	Y	Y	Y	Y	8
Osayem J,et al	Y	Y	N	Y	Y	Y	Y	Y	Y	8
Yenegeta Z,et al	Y	Y	Y	Y	Y	Y	Y	Y	Y	9
Alemu D ,et al	Y	Y	N	Y	Y	Y	Y	Y	Y	8
Ibanga, A,et al	Y	Y	Y	Y	Y	Y	Y	Y	Y	9
Ogbonnaya C,et al	Y	Y	Y	Y	Y	Y	Y	Y	Y	8

Q1-Q9 represents questions used to assess the quality of included studies, which are listed below

Q1. Was the sample frame appropriate to address the target populations?

Q2. Were the study participants sampled in appropriate way?

Q3. Was the sample size adequate?

Q4. Were the study subjects and setting described in details?

Q5. Was the data analysis conducted with sufficient coverage of the identified sample?

Q6. Was a valid method used in the identification of conditions?

Q7. Was the condition measured in a standard, reliable way for all participants?

Q8. Was there an appropriate statistical analysis?

Q9. Was the response rate adequate, and if not, was the low response rate managed appropriately?

Key: N = No; NA=Not applicable; U= Unclear; Y= Yes
